# Diverse antiepileptic drugs increase the ratio of background synaptic inhibition to excitation and decrease neuronal excitability in neurones of the rat entorhinal cortex *in vitro*

**DOI:** 10.1016/j.neuroscience.2010.02.021

**Published:** 2010-05-05

**Authors:** S.D. Greenhill, R.S.G. Jones

**Affiliations:** Department of Pharmacy and Pharmacology, University of Bath, Claverton Down, Bath, BA2 7AY, UK

**Keywords:** entorhinal cortex, background excitation, background inhibition, voltage fluctuations, neuronal excitability, antiepileptics, aCSF, artificial cerebro-spinal fluid, AED, antiepileptic drugs, AMPAr, AMPA-receptors, AP, action potential, E_bg_, background excitation, EC, entorhinal cortex, GABA_A_r, GABA_A_-receptors, GABA_B_r, GABA_B_-receptors, I_bg_, background inhibition, IEI, interevent interval, KS, Kolmogorov–Smirnov, NBQX, 6-nitro-7-sulphamoylbenzo[f]quinoxalone-2,3-dione disodium, NMDA, N-methyl-d-aspartate, NMDAr, NMDA-receptors, PDC, l-trans-pyrrolidine,2–4,dicarboxylate, sEPSC, spontaneous excitatory postsynaptic current, sIPSC, spontaneous inhibitory postsynaptic current, TTX, tetrodotoxin, VGCC, voltage gated calcium channels, VGNC, voltage-gated Na-channels, VmD, membrane potential distribution, 2-AP5, 2-amino-5-phosphonopentanoic acid, 4-AP, 4-aminopyridine

## Abstract

Although most anti-epileptic drugs are considered to have a primary molecular target, it is clear that their actions are unlikely to be limited to effects on a single aspect of inhibitory synaptic transmission, excitatory transmission or voltage-gated ion channels. Systemically administered drugs can obviously simultaneously access all possible targets, so we have attempted to determine the overall effect of diverse agents on the balance between GABAergic inhibition, glutamatergic excitation and cellular excitability in neurones of the rat entorhinal cortex *in vitro*. We used an approach developed for estimating global background synaptic excitation and inhibition from fluctuations in membrane potential obtained by intracellular recordings. We have previously validated this approach in entorhinal cortical neurones [Greenhill and Jones (2007a) Neuroscience 147:884–892]. Using this approach, we found that, despite their differing pharmacology, the drugs tested (phenytoin, lamotrigine, valproate, gabapentin, felbamate, tiagabine) were unified in their ability to increase the ratio of background GABAergic inhibition to glutamatergic excitation. This could occur as a result of decreased excitation concurrent with increased inhibition (phenytoin, lamotrigine, valproate), a decrease in excitation alone (gabapentin, felbamate), or even with a differential increase in both (tiagabine). Additionally, we found that the effects on global synaptic conductances agreed well with whole cell patch recordings of spontaneous glutamate and GABA release (our previous studies and further data presented here). The consistency with which the synaptic inhibition:excitation ratio was increased by the antiepileptic drugs tested was matched by an ability of all drugs to concurrently reduce intrinsic neuronal excitability. Thus, it seems possible that specific molecular targets among antiepileptic drugs are less important than the ability to increase the inhibition:excitation ratio and reduce overall neuronal and network excitability.

Antiepileptic drugs (AED) comprise a heterogeneous group of agents with diverse mechanisms of action. Many have multiple effects on neuronal activities and overall mechanisms of action are a source of debate. As a generalization, antiepileptic drugs aim to modify the dynamic balance between inhibition and excitation in neuronal circuits by targeting membrane ion channels, transmitter receptors and metabolic pathways. Attempts to categorize AED according to their molecular targets have generally assigned them to three main groups; those that block voltage gated ion channels (Na and Ca), and those that either enhance GABAergic inhibition (by altering GABA synthesis/breakdown or potentiating GABA_A_-receptor (GABA_A_r) activity) or reduce glutamatergic excitation (blocking glutamate receptors, reducing glutamate release) (e.g. Rogawski and Loscher, 2004; Landmark, 2007; White et al., 2007). However, AED are administered systemically and access all elements of the neuronal networks. Thus, the specific molecular targets of the drugs may be less important in controlling epilepsy than the overall global effects on network excitability.

Recently, using whole-cell patch clamp recordings of spontaneous excitatory postsynaptic currents (sEPSCs) and spontaneous inhibitory postsynaptic currents (sIPSCs), we have shown that a number of clinically effective drugs can modify the spontaneous release of glutamate and GABA in the rat entorhinal cortex (EC). Phenytoin and lamotrigine both elicited a presynaptic modulation of glutamate and GABA release, decreasing the former, but concurrently increasing the latter (Cunningham et al., 2000; Cunningham and Jones, 2000). Neither effect was related to drug action at voltage-gated Na-channels (VGNC). Valproate also decreased spontaneous glutamate release, but this effect was likely to be due to blockade of VGNC in presynaptic axons (Cunningham et al., 2003). Unlike phenytoin and lamotrigine, valproate also weakly decreased GABA release (again by blocking VGNC), but at the same time prolonged the decay time of sIPSCs, probably by a postsynaptic effect. In other studies we have shown that gabapentin, pregabalin (Cunningham et al., 2004) and felbamate (Yang et al., 2007) also decrease glutamate release, albeit by different mechanisms, but have little effect on GABA release (Jones, unpublished observations; current paper).

The overall significance of the effects on spontaneous glutamate and GABA release in the mechanism of action of AED is unclear as yet. However, continuous spontaneous release of both glutamate and GABA resulting from action potentials within network interconnections, and also in the form of mono-quantal, activity-independent release (miniature events), is a characteristic of cortical neurones. An increasing school of thought suggests that such background activity strongly modifies signal detection. Thus, the relative level of inhibitory and excitatory background activity is a reflection of ongoing network activity and is instrumental in determining the excitability of any given neurone (Pare et al., 1998; Stevens and Zador, 1998; Destexhe and Pare, 1999; Ho and Destexhe, 2000; Rudolph and Destexhe, 2001; Stacey and Durand, 2000; Fellous et al., 2003; Shu et al., 2003a,b). We have previously speculated that the balance of spontaneous inhibition and excitation is a determinant of excitability in EC neurones (Jones and Woodhall, 2005).

Our patch clamp studies clearly show that there is a diversity of effects of AED on background transmitter release, but again we are looking at target specific effects. We believe that is essential to determine the relative change in global background synaptic activities and how this relates to neuronal excitability. Whole cell patch clamp studies are limited in several respects. Experimental conditions are tailored towards recording of either excitatory or inhibitory events in isolation. Recording both inhibitory and excitatory activity simultaneously is possible, but this places significant limits on recording quality. Soma recordings may not adequately detect distal dendritic events that contribute to overall cellular activity. Manipulations aimed at improving space-clamp and, thus, recording resolution (blockade of VGNC and some K-channels) mean that realistic estimation of postsynaptic cellular excitability is impossible. To overcome these problems and obtain estimates of background synaptic activity we have begun to use an approach described by Rudolph et al. (2004), whereby estimates of global background synaptic excitation (E_bg_) and inhibition (I_bg_) are derived simultaneously from fluctuations in membrane voltage obtained from sharp electrode intracellular recordings. This has been termed the membrane voltage distribution (VmD) approach. Intracellular recordings preserve the intracellular milieu and thus also allow for estimates of cellular excitability. We have validated this approach in a previous paper and shown that it can usefully be applied to intracellular recordings taken from EC neurones in slices (Greenhill and Jones, 2007a). Using the VmD approach, we have now determined the ratio of background inhibition to excitation in layer III neurones in the EC. We find that this global ratio is consistently altered in favour of increased inhibition by all AED tested, regardless of their specific molecular targets. At the same time the drugs elicit a consistent decline in neuronal excitability. Some of these results have been reported in abstracts (Greenhill and Jones, 2007b; Greenhill et al., 2008, 2009).

## Experimental procedures

### Slice preparation

All experiments were performed in accordance with the U.K. Animals (Scientific Procedures) Act 1986, European Communities Council Directive 1986 (86/609/EEC) and the University of Bath ethical review document. The number of animals used was kept to a minimum and every precaution was taken to minimize any suffering and stress inflicted. EC slices were prepared from male Wistar rats (60–70 g, bred in-house) as described previously (Jones and Heinemann, 1988). They were anaesthetized with ketamine (120 mg/kg; Fort Dodge Animal Health Ltd., Southampton, UK) and xylazine (8 mg/kg; Bayer plc, Bury St Edmunds, UK) administered by intra-muscular injection. After decapitation the whole brain was quickly removed and immersed in chilled (4 °C) oxygenated (95% O_2_*/*5% CO_2_) artificial cerebrospinal fluid (aCSF) containing ketamine (10 μM). The use of ketamine as an anaesthetic, and in the cutting solution greatly improves the viability and durability of the slices, presumably by blocking excitotoxic effects of NMDAr activation during preparation. Residual blockade of the NMDAr rapidly disappears during storage and recovery evidenced by the ability to record pronounced NMDAr mediated effects during subsequent recordings (e.g. Cunningham et al., 2000; Dhillon and Jones, 2000; Woodhall et al., 2001) Slices (400 μm) were cut with a Vibroslice (Campden Instruments, Loughborough, UK) and transferred to a holding chamber containing aCSF (without ketamine) at room temperature. After a recovery period of at least 1 h, individual slices were transferred to an interface chamber perfused with oxygenated aCSF (1.5 ml/min) maintained at 32±0.5 °C for recording. ACSF consisted of (in mM): NaCl (126), KCl (3.75), MgSO_4_ (1.5), NaHCO_3_ (23), NaH_2_PO_4_ (1.4), CaCl_2_, (2) and d-glucose (10), pH 7.4 at recording temperature.

### VmD experiments

Intracellular voltage recordings were made with sharp electrodes filled with potassium acetate (3 M) from pyramidal neurones in layer III of the medial EC using an Axoprobe 1A amplifier (Molecular Devices, Sunnyvale, CA, USA) in bridge mode. Data were acquired using a Digidata 1200 and Axoscope software. Typically (*n*=6), these neurones had a resting potential of −72.2±0.5 mV, input resistance of 79±6 mΩ and action potential amplitude (from threshold) and half-widths of 72.1±1.6 mV and 0.32±0.01 ms, respectively. In an initial series of experiments synaptic responses were evoked via a bipolar stimulating electrode placed in the lateral EC. In six neurones excitatory postsynaptic potentials mediated by AMPA receptors (AMPAr) were isolated by blockade of NMDA-receptors (NMDAr, 2-AP5), GABA_A_r (bicuculline) and GABA_B_-receptors (GABA_B_r, CGP 55845A). The mean reversal potential of AMPAr-mediated responses (+6.6±2.0 mV) was determined by recording excitatory postsynaptic potentials at different membrane potentials. Similar experiments (*n*=6) were performed, substituting an AMPAr antagonist (NBQX) for the GABA_A_r-antagonist, in the blocking cocktail, to determine the reversal potential (−66.7±1.0 mV) of GABA_A_r-mediated inhibitory postsynaptic potentials.

At regular intervals throughout the recordings, following an equilibration time of 5–10 min after stabilization of resting potential, estimates of E_bg_ and I_bg_ were made using the VmD method derived by Rudolph et al. (2004), and as previously described (Greenhill and Jones, 2007a). Briefly, neurones were depolarised (for 15–20 s) by injection of two levels of known positive current (I_ext1_ and I_ext2_) via the recording electrode. The values of the currents differed from neurone to neurone, but were maintained the same throughout any individual experiment. I_ext2_ was chosen to elicit a depolarization to within 1–2 mV of action potential threshold and I_ext1_ was adjusted to depolarize the neurone to about half way between I_ext2_ and resting membrane potential. Membrane potential fluctuations at these two levels were fitted to Gaussian distributions (using Prism 4 software, GraphPad, San Diego, CA, USA) and the mean and variance of the membrane potential determined. Leak conductance in each neurone was calculated from the ohmic response produced by a small (0.1 nA, 100 ms) hyperpolarizing current, injected at resting membrane potential. These parameters, together with the mean reversal potentials derived from the preliminary experiments, allowed us to use the VmD relationship (equation 11 in Rudolph et al., 2004) to quantify background inhibitory and excitatory conductances resulting from global network input to individual neurones.

Cellular excitability was determined by injecting depolarizing current pulses at resting potential during intervals between E_bg_ and I_bg_ estimates. Firstly, action potential (AP) thresholds were determined using brief incremental peri-threshold injections of depolarizing current (0.1–1.0 nA, 50 ms) via the recording electrode, with firing threshold measured with respect to resting membrane potential. Secondly, trains of action potentials were elicited by longer, supra-threshold current steps (0.4–1.5 nA, 250 ms), and the number of spikes per step depolarization determined. Action potential amplitudes (from rest) and half-widths were also analyzed.

Statistical analysis (paired *t*-test or one-way ANOVA) was performed with Prism 4 software. All values are expressed as mean±SEM.

### Whole-cell patch clamp recordings

We had not previously reported the effects of some of AED tested on sEPSCs and sIPSCs. To provide a more complete correlation between these effects and the corresponding VmD estimations of E_bg_ and I_bg_, we tested the effects of felbamate and gabapentin on sIPSCs, and tiagabine on both sIPSCs and sEPSCs.

Slices were prepared as described above. For recording sEPSCs, patch pipettes were filled with a Cs-gluconate based solution containing (in mM) d-Gluconate (100), HEPES (40), QX-314 (1), EGTA (0.6), MgCl_2_ (5), TEA-Cl (10), phosphocreatinine (5); ATP-Na (4) and GTP-Na (0.3). To record sIPSCs the patch solution contained CsCl (100), HEPES (40), QX-314 (1), EGTA (0.6), TEA-Cl (10), MgCl_2_ (5), ATP-Na (4) and GTP-Na (0.3). Solutions were adjusted to 275 mOsmol, and to pH 7.3 with CsOH. Whole-cell voltage clamp recordings (holding potential −60 mV unless otherwise stated) were made from neurones in layer III of the medial division of the EC, using an Axopatch 200B amplifier. Signals were filtered at 2 kHz and digitized at 20 kHz. Series resistance compensation was not employed, but access resistance (10–30 MΩ) was monitored at regular intervals throughout each recording and cells were discarded from analysis if it changed by more than ±10%.

To compare sPSCs we measured amplitudes, inter-event intervals (IEI), 10–90% rise times, and total decay times. In some cases, we found complex effects on sPSCs (e.g. decrease in frequency, increase in decay time) so to gain an overall picture of the effects of drugs on the level of spontaneous inhibition or excitation we estimated total charge transfer associated with sIPSCs and sEPSCs. This is calculated by measuring the area of sIPSCs or sEPSCs, and is directly proportional to the amplitude multiplied by the decay time (Hollrigel and Soltesz, 1997). We determined charge transfer associated with sPSCs in a set time period of 2 min in control and in the presence of the drug. Mean values for all parameters of sPSCs were compared using a paired *t*-test, with significance set at *P*<0.05. For additional comparison of IEI we used the non-parametric Kolmogorov–Smirnov test (KS) applied to cumulative probability distributions. When pooling data for this comparison we included a minimum of 200 events from each neurone in the control situation and an equal number of events in the presence of the drug. Significance was set at *P*<0.01.

### Materials

The following drugs were used: felbamate (2-phenyl-1,3-propanediol dicarbamate, Sigma-Aldrich, UK), gabapentin (1-(aminomethyl)cyclohexaneacetic acid, a gift from Pfizer Global R and D, Ann Arbor, MI, USA), lamotrigine (6-(2,3-dichlorophenyl)-1,2,4-triazin-3,5-diamine, Tocris, UK), phenytoin (5,5-diphenylhydantoin, Sigma-Aldrich, UK), sodium valproate (Sigma-Aldrich, UK), tiagabine (Tocris, UK), NBQX (6-nitro-7-sulphamoylbenzo[f]quinoxalone-2,3-dione disodium, Tocris, UK), 2-AP5 (2-amino-5-phosphonopentanoic acid, Tocris, UK), bicuculline methochloride (Tocris, UK), PDC (l-trans-pyrrolidine,2–4,dicarboxylate, Tocris, UK), CGP 55845A ((2S)-3-[[(1S)-1-(3,4-dichlorophenyl)ethyl]amino-2-hydroxypropyl](phenylmethyl)phosphinic acid, a gift from Novartis International AG, Basel, Switzerland), fluoxetine (Ascent Scientific, UK), clozapine (Ascent Scientific, UK), 4-AP (4-aminopyridine, Sigma-Aldrich, UK), TTX (Alamone Labs, Israel). All drugs were applied by bath perfusion. QX-314 (Tocris, UK) was included in patch pipette solutions. We based the concentrations of AED and other drugs on those that have been employed in a large variety of other pharmacological experiments in the literature including our own (e.g. Cunningham et al., 2000, 2003, 2004; Cunningham and Jones, 2000; Yang et al., 2007). These have been based as far as possible on the therapeutic range of brain concentrations described in the literature (e.g. see Rogawski and Porter, 1990). In the main, we kept our test concentrations at the lower end of the range at which significant pharmacological actions might be expected without direct effects on excitability, as we wished to correlate changes in synaptic activity with excitability changes. We accept that higher or lower concentrations may have different effects on background excitation and inhibition to those reported here.

## Results

The experiments described below involved recordings (intracellular and patch clamp) obtained from a total of 96 neurones of the medial EC. In sharp electrode recordings we selected neurones in layer III as pyramidal, based on firing characteristics, using criteria established from a previous study using sharp electrode recording and biocytin fills (Dhillon and Jones, 2000). In whole-cell patch recordings, neurones were visually identified in slices using IR-DIC, and those with clear pyramidal morphology selected for recording. We cannot guarantee that every neurone recorded from was a pyramid but it is likely that the vast majority is.

In VmD experiments (*n*=48) the mean control values of I_bg_ in layer III neurones was 6.3±0.9 nS. E_bg_ was considerably lower at 1.7±0.3 nS and the mean I:E ratio was 4.0±0.3. This predominance of background inhibition over excitation is similar to that reported previously *in vitro* (Rudolph et al., 2004; Greenhill and Jones, 2007a) and also in similar estimates *in vivo* (Rudolph et al., 2007). There was a marked variation in control values from neurone to neurone, but I_bg_ was always higher than E_bg_.

### Effects of AED

#### Phenytoin

**Fig. 1:**
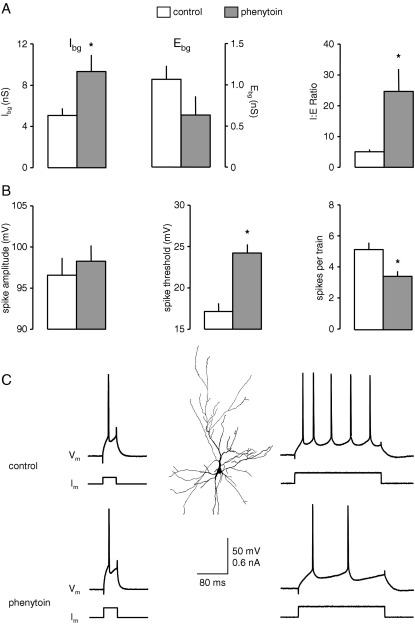
Effect of phenytoin on background synaptic conductances and excitability. (A) Phenytoin concurrently increased background inhibition (I_bg_) but decreased background excitation (E_bg_). The net result of this was a fivefold increase in the I:E ratio. (B) Measures of cellular excitability indicate that the increase in I:E ratio was accompanied by reduced excitability. Thus, spike threshold was elevated and repetitive firing during long depolarizing pulses was reduced. Spike amplitude was unchanged. (C) Intracellular recordings from one neurone illustrating the excitability change. The top line shows typical responses evoked by depolarizing current in layer III pyramidal neurones (shown by the reconstructed neurone following a biocytin fill). The bottom line shows that the current required to elicit a spike at threshold was larger in the presence of the drug. When a supra-threshold, long duration pulse was used to elicit repetitive firing, the number of spikes evoked was reduced in the presence of phenytoin. Asterisks indicate a significant difference compared to control values (*P*<0.05, paired *t*-test).

The effects of phenytoin (20 μM) were tested on six neurones and the results are summarized in Fig. 1. In control recordings the mean value of I_bg_ was 5.1±0.7 nS, and E_bg_ was almost fivefold lower at 1.1±0.2 nS, resulting in a mean I:E ratio of 5.1±0.7 (Fig. 1A). In the presence of phenytoin, I_bg_ increased to 9.3±1.6 nS (*P*<0.05, paired *t*-test). Concurrently, E_bg_ decreased by 50±9% to 0.6±0.2 nS, although this just failed to reach significance. However, the I:E ratio showed a pronounced increase in favour of inhibition, to 24.6±7.3 (548±212%; *P*<0.05; Fig. 1A). These effects are in good agreement with our previous patch clamp experiments in EC neurones. In these we showed that phenytoin, at the same concentration employed here, acted presynaptically to increase the spontaneous release of GABA, but to decrease the release of glutamate (Cunningham et al., 2000; Yang et al., 2007).

Concurrent with the increase in I:E ratio induced by phenytoin, the drug also significantly altered neuronal excitability. Thus, in the six neurones tested, AP threshold increased from 17.1±1.0 mV positive to E_M_ to 24.3±1.0 mV (*P*<0.05, *t*-test). The number of APs generated during a long depolarizing current step fell from 5.3±0.5 to 3.5±0.3 (*P*<0.001, *t*-test). However, there was no change in action potential amplitude (96.5±2.1 versus 98.2±1.9 mV) or half width (0.41±0.03 versus 0.39±0.01 ms). Additionally, during evoked spike trains, phenytoin did not significantly change the amplitude of the final action potential in each train, or the relative amplitudes of the first and last spikes (not shown). The results in the six neurones tested are summarized in Fig. 1B. Fig. 1C shows responses of one neurone to depolarizing current pulses and illustrates the increased amplitude of a short current pulse to elicit a spike at threshold, and the decreased number of spikes in the train evoked by the long, supra-threshold step. Thus, in summary, phenytoin induced an increase in I:E ratio, which was concurrent with a decreased neuronal excitability, but without directly affecting AP generation.

#### Lamotrigine

Results with lamotrigine (20 μM) were very similar to those noted with phenytoin. During application of the drug, I_bg_ increased progressively from a control value of 5.3±2.0 to 13.2±2.6 nS after 15 min (*P*<0.05). Concurrently, E_bg_ decreased from 1.5±0.4 to 1.1±0.4 nS. The large variation in E_bg_ in this sample of neurones meant that the difference just failed to reach significance. Noticeable, however, was the marked change in I:E ratio. From a control value of 4.0±0.7, this increased to 17.5±5.0 nS (*P*<0.05) a mean increase of 434±112% in favour of inhibition in the six neurones tested. Again, the results obtained with lamotrigine in the VmD estimates agree with those obtained in whole cell patch clamp recordings, where we showed a decrease in frequency of spontaneous AMPAr mediated sEPSCs concurrent with an increase in GABA_A_r-mediated sIPSCs (Cunningham and Jones, 2000).

The pronounced increase in I:E ratio was paralleled by a decline in neuronal excitability. Spike threshold increased from 25.3±3.3 mV in control to 29.1±3.8 mV after 15 min (*P*<0.05). The number of spikes generated by a 250 ms depolarizing step decreased from 5.3±0.8 to 1.3±0.6 (*P*<0.05). Spike amplitude (94.2±0.7 versus 95.5±0.9 mV) and half width (0.37±0.01 versus 0.4±0.02 ms) were unaltered.

Thus, as with phenytoin, an overall increase in global inhibition by lamotrigine was accompanied by a decrease in neuronal excitability, with no apparent deleterious effects on spike generation. We tested a higher concentration (200 μM) of lamotrigine in two neurones. This resulted an increase in mean I_bg_ from 5.5 to 27.7 nS and a decrease in E_bg_ from 1.6 to 0.8 nS, with a huge concomitant increase in I:E from 3.6 to 29.5. However, although spike threshold (27.2 versus 31 mV) and spike frequency (3.7 versus 1.0) were both clearly reduced, there was also a substantial decrease in spike amplitude (94.7 versus 77.7 mV) and increase in half-width (0.31 versus 0.52 ms) as well in these two neurones. Thus, although the higher concentration of the drug had qualitatively similar effects in VmD studies, it was clearly also affecting spike generation.

#### Carbamazepine

Carbamazepine is generally categorized alongside phenytoin and lamotrigine as a front-line treatment for partial and generalised tonic-clonic seizures, with VGNC as a primary molecular target (Rogawski and Loscher, 2004; White et al., 2007). In VmD studies it had a very similar effect to the other two drugs. Thus, it elevated I_bg_ from 4.7±1.8 to 11.2±2.6 nS after 15 min of perfusion (*P*<0.05, *n*=4), while E_bg_ was decreased from 2.1±0.4 to 1.3±0.8 nS, although the latter was not significant. Overall, the I:E ratio rose from 3.8±1.1 to 11.9±1.9 (*P*<0.05). Measures of cellular excitability also showed the same changes seen with phenytoin and lamotrigine with an increase in spike threshold and a decrease in the number of spikes evoked during prolonged depolarization, but no alteration in spike parameters (not shown).

#### Valproate

**Fig. 2:**
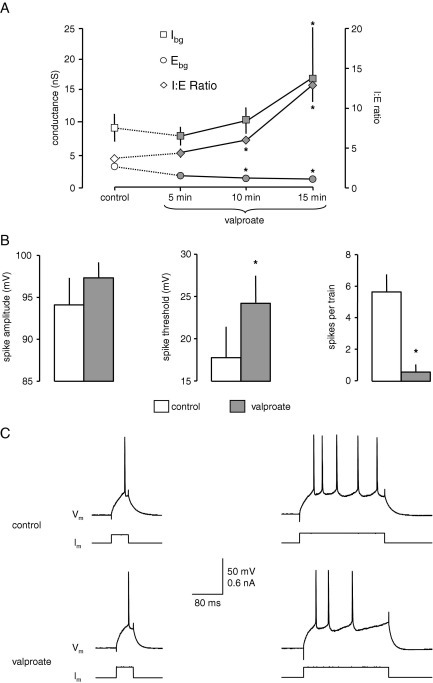
Valproate also increases I:E ratio. (A) Summary of the time course of changes in six neurones over 15 min. There was an initial slight fall in I_bg_, with a subsequent elevation. E_bg_ was progressively reduced throughout, and overall there was a progressive increase in I:E ratio. (B) and (C) show that this change was accompanied by similar changes in excitability to those seen with phenytoin and lamotrigine. Asterisks indicate a significant difference compared to control values (*P*<0.05, paired *t*-test).

Valproate is generally considered to be a broad spectrum AED, used to treat generalized (tonic-clonic and absence) and partial seizures. During perfusion with valproate (500 μM, *n*=6) we recorded an elevation in background inhibition, with I_bg_ increasing from 10.2±2.0 to 23.1±4.4 nS after 15 min (*P*<0.05). Conversely, there was a significant decline in E_bg_ from 2.8±0.4 to 1.7±0.3 nS (*P*<0.05). These concurrent changes again resulted in a substantial change in the I:E ratio, from 3.6±0.4 to 14.1±3.0 (*P*<0.05). In these experiments we monitored the time course of the changes in background conductances, and this is illustrated in Fig. 2A. Interestingly, after 5 min, there was a slight decline in I_bg_. However, this was not significant and thereafter a progressive rise was recorded. In contrast, E_bg_ declined steadily throughout the 15-min recording period.

In previous whole cell patch clamp experiments (Cunningham et al., 2003) we found that valproate decreased the frequency of sEPSCs and also that of sIPSCs, albeit more weakly, contrasting with lamotrigine and phenytoin, which decreased the former but increased the latter. We showed that both effects were probably due to blockade of VGNC in presynaptic axons/terminals. At first sight this is at odds with the observation in the current experiments that the drug increased I_bg_. However, although valproate decreased the frequency of sIPSCs it prolonged their decay time without effect on amplitude (Cunningham et al., 2003). To look at this in more detail we have reanalysed the data from these experiments to calculate the inhibitory charge transfer in the presence and absence of valproate. This analysis gave a mean inhibitory charge transfer of 1352.8±331 pC in control conditions but, despite the reduction in frequency, 1727±391 pC in the presence of valproate. Thus, the increased duration of sIPSCs induced by the drug appears to compensate for the reduced number of events and would be in line with the increase in global background inhibition over excitation recorded in our VmD analysis.

Valproate, like phenytoin and lamotrigine, also depressed neuronal excitability Pooled data from the six neurones tested are shown in Fig. 2B and responses from one neurone in Fig. 2C. Action potential threshold increased from 17.8±3.6 to 24.2±3.2 mV after 15 min (*P*<0.05), while the number of spikes generated by the long depolarizing pulse fell from 5.7±1.1 to 0.6±0.5 (*P*<0.05). Interestingly, spike amplitude was not affected, and actually increased slightly (though not significantly) from 94.2±3.3 to 97.3±2 mV. Spike half-width was unaltered (0.36±0.01 versus 0.38±0.02 ms). In whole cell patch clamp experiments the decrease in sIPSC and sEPSC frequency were obviated by prior application of TTX suggesting that valproate was blocking VGNC and spike generation in presynaptic axons. Thus, it may be that presynaptic action potential generation may be more sensitive to valproate than postsynaptic.

#### Gabapentin

Gabapentin is one of the newer generation of AEDs, and is licensed for use in partial epilepsies with or without secondary generalization. It is a GABA analogue originally designed to act as a brain-penetrant GABAr-agonist but the consensus of opinion is that it generally has little effect at GABA receptors or other elements of GABAergic systems (Taylor et al., 1998). It has been shown to a interact with the α_2_δ-auxiliary subunit of voltage gated calcium channels (VGCC), and in whole cell patch clamp studies we found that gabapentin reduced both evoked and spontaneous release of glutamate at EC synapses (Cunningham et al., 2004; Yang et al., 2007). This appeared to be due partly to blockade of P/Q-type VGCC, and partly due to an effect on glutamate release downstream of Ca-entry.

We have now tested the effect of gabapentin on GABA_A_r-mediated sIPSCs in 6 EC neurones using whole cell patch clamp. In these neurones sIPSCs had a mean IEI of 155±31 ms (corresponding to a frequency of 7.1±2.1 Hz) in control conditions. Gabapentin (25 μM) slightly (but not significantly) reduced sIPSC frequency to 6.1±2.3 Hz (IEI 171±38 ms). Likewise, the drug had little effect on peak amplitude of sIPSCs (27.6±1.7 versus 28.8±2.1 pA), 10–90% rise time (2.2±0.5 versus 2.4±0.3 ms) or total decay time (17.1±1.9 versus 18.6±2.2 ms). Overall, inhibitory charge transfer was very similar whether the drug was present (779.4±224 pC) or absent (687±271 pC).

**Fig. 3:**
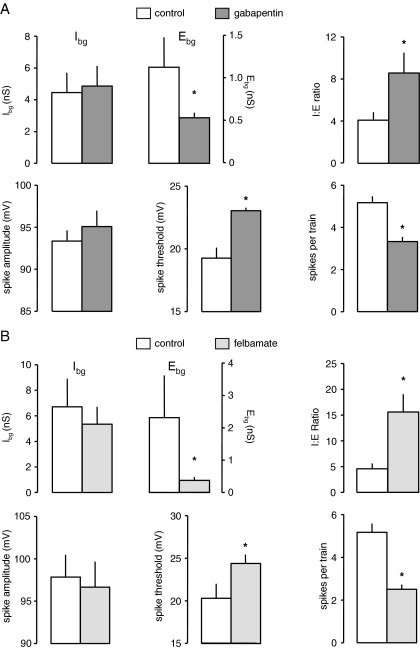
Summary data for gabapentin and felbamate. (A) Gabapentin decreased E_bg_. Despite a lack of effect on I_bg_, the drug, nevertheless, increased I:E ratio. Concurrently excitability of neurones was reduced. (B) Felbamate resembled gabapentin in that it increased I:E ratio primarily as a result of decreasing E_bg_. It also significantly reduced excitability. The asterisks indicate significant differences compared to control values. Asterisks indicate a significant difference compared to control values (*P*<0.05, paired *t*-test).

So in whole cell clamp studies gabapentin decreased spontaneous glutamate release but had no effect on GABA. In agreement with these data, and in contrast to the other AED described above, gabapentin (25 μM) had little effect on I_bg_ in six neurones where we conducted VmD analysis. In control recordings I_bg_ was 4.5±1.3 nS and this was unaltered after 15 min perfusion with gabapentin (4.9±1.3 nS). However, E_bg_ was more than halved (1.2±0.3 to 0.5±0.1 nS, *P*<0.05, *t*-test), again in good agreement with our patch clamp studies (Cunningham et al., 2004). Thus, although background inhibition *per se* was largely unaltered by gabapentin, the slight increase, together with the decline in background excitation meant that I:E ratio was again significantly increased from 4.1±0.7 to 8.6±1.9 in favour of inhibition (Fig. 3A; *P*<0.05, *t*-test).

The shift in I:E ratio by gabapentin was accompanied by changes in excitability, as with the other AEDs (Fig. 3A). Action potential threshold increased from 19.3±0.8 to 23.0±0.3 mV after 15 min (*P*<0.05, *t*-test). The decrease in number of spikes evoked by a depolarizing pulse was not quite as marked as with other drugs tested (5.2±0.3 to 3.3±0.2), but was, nevertheless, still significant (*P*<0.05, *t*-test). Spike amplitude was unaltered (Fig. 3A).

#### Felbamate

Felbamate was the first of the new generation of AED and was first introduced in 1993 for the treatment of a wide spectrum of seizures including partial, primary and secondarily generalized seizures, and Lennox–Gastaut syndrome. However, hepatic and haematological toxicity mean its use is now limited to treatment of the latter and other refractory epilepsies.

We have previously reported that low concentrations of felbamate reduce the frequency of sEPSCs in whole cell patch clamp recordings by around 50% (Yang et al., 2007). The drug has repeatedly been shown to be an antagonist at NMDAr, and our data suggested that its effects on spontaneous glutamate release were likely to be due to blockade of presynaptic facilitatory NMDA autoreceptors (see Berretta and Jones, 1996; Woodhall et al., 2001). We have tested the effect of felbamate (100 μM) in VmD studies (*n*=6). In line with the whole cell patch studies E_bg_ was substantially reduced from 2.3±1.3 to 0.4±0.1 nS (Fig. 3B).

Previous reports have suggested felbamate can enhance GABA_A_r mediated currents in cortical neurones (Kume et al., 1996; Rho et al., 1997), although studies in *xenopus* oocytes indicated a positive or negative modulation, depending on receptor subunit composition (Simeone et al., 2006). However, in our VmD experiments, I_bg_ was slightly, but not significantly reduced in the presence of felbamate (6.7±2.2 versus 5.4±1.4 nS; Fig. 3B). To clarify and complement this observation we conducted whole cell patch clamp studies on sIPSCs in six EC neurones. Application of felbamate (100 μM) caused a small, but non-significant decrease in frequency of sIPSCs (IEI 169±36 versus 201±54 ms). In addition, the mean amplitude (31.2±2.1 versus 28.9±2.6 pA), rise (1.9±0.5 versus 1.8±0.3 ms) and decay time (15.4±1.8 versus 16.8±2.0 ms) of these events were also unaltered.

Thus, the patch clamp studies are in agreement with the lack of change in I_bg_. However, the concurrent decline in E_bg_ resulted in a cumulative change in I:E ratio from 4.6±1.0 to 15.6±3.5; *P*<0.05, *t*-test, favouring background inhibition (Fig. 3B). Again, this relative change in I_bg_ and E_bg_ proved to be a good predictor of changes in neuronal excitability (Fig. 3B). Thus, spike firing threshold was raised from 20.3±1.7 to 24.4±1.0 mV (*P*<0.05) by the addition of felbamate and the number of spikes generated by the 250 ms depolarizing pulse fell by about 50% (5.2±0.4 to 2.5±0.2; *P*<0.05). Although, very high concentrations of felbamate have also been reported to inhibit VGNC (Taglialatela et al., 1996) it had no effect on either spike amplitude (97.9±2.6 versus 96.7±3.0 mV) or half-width (0.42±0.02 versus 0.40±0.03 ms) in our studies.

#### Tiagabine

**Fig. 4:**
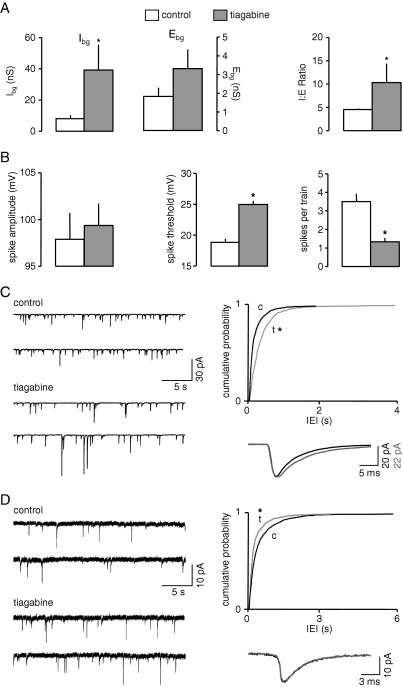
Summary of VmD and whole cell patch experiments with tiagabine. (A) The drug increased both E_bg_ and I_bg_, but a more pronounced effect on the latter resulted in an overall increase in I:E ratio. (B) This was accompanied by a similar decrease in excitability to that seen with other AED. (C) Recordings of sIPSCs in one neurone show a decrease in frequency accompanied by an increase in amplitude of events. The cumulative probability curves (pooled data from six neurones) show the distribution of interevent intervals shifted to right of control (c) in the presence on tiagabine (t, grey line). Averaged sIPSCs (60 each in control and drug) in one neurone are also shown. These are scaled to the same peak amplitude and show that the decay is prolonged by tiagabine (grey line). (D) sEPSCs recorded in one neurone show a clear increase in frequency in the presence of tiagabine. The cumulative probability curve of interevent intervals is shifted to the left of control (c) in the presence of tiagabine (t). The averaged sEPSCs in one neurone (90 each in control and drug) overlap almost perfectly showing that the primary effect was on frequency. The asterisks in (A) and (B) indicate significance at *P*<0.05 compared to control values, assessed by paired *t*-tests, and in (C) and (D) at *P*<0.01 assessed by KS.

Tiagabine is a selective GABA-reuptake inhibitor structurally related to nipecotic acid. It has been successfully used as add-on therapy in the treatment of partial epilepsies (Bauer and Cooper-Mahkorn, 2008). VmD estimates were obtained from a total of six neurones. Perhaps unsurprisingly, tiagabine (4 μM) caused a sixfold rise in I_bg_ from a control level of 8.1±2.1 to 48.8±16.8 nS (*P*<0.05). Interestingly, however, E_bg_ was also increased but to a much lesser extent, from 1.8±0.4 to 3.9±1.0 nS (*P*<0.05). Nevertheless, although E_bg_ was more than doubled, the overall change in both conductances again shifted the I:E ratio heavily in favour of inhibition, from 4.5±0.2 to 12.5±4.4 (*P*<0.05). The data are summarized in Fig. 4A.

We have also conducted parallel studies of tiagabine on sIPSCs and sEPSCs to determine if the global changes determined from VmD estimates was paralleled by changes in spontaneous glutamate and GABA release. Tiagabine (4 μM, *n*=6) had complex and quite variable effects on sIPSCs. Overall IEI increased from 180±52 to 256±45 ms reflecting a fall in mean frequency from 8.4±2.2 to 5.8±2.3 Hz. KS analysis showed the change in IEI to be significant (*P*<0.01), although the change in frequency (paired *t*-test) was not. Recordings from one neurone are shown in Fig. 4C, together with the cumulative probability analysis of pooled data from the six neurones tested. It should be noted that, in the population of neurones tested, four showed a clear decrease in frequency whereas there was no change in one and a slight increase in another. In addition to the overall frequency change, mean sIPSC amplitude increased significantly (from 16.7±1.7 to 23.8±2.7 pA) and this was consistent in all six neurones. Concurrently, the drug consistently increased the total decay time of sIPSCs from 28.6±0.7 to 37.3±2.4 ms (*P*<0.05). Averaged sIPSCs from one neurone are illustrated in Fig. 4C to show the prolonged decay time. To make an estimate of the overall effect of tiagabine on spontaneous GABA release, we calculated the total charge transfer associated with sIPSCs. The mean inhibitory charge transfer in control was 548.3±161.7 pC, and this increased to 767.8±247.5 pC in the presence of the drug, a mean increase of 148±36%. However, the variability between cells meant that this did not reach significance.

Tiagabine also had noticeable effects on sEPSCs, and the data are summarized in Fig. 4D. The most pronounced effect was an increase in frequency. Thus, in five neurones, IEI decreased from 432±128 to 204±60 ms during application of tiagabine (4 μM) reflecting an increase in frequency from 3.4±0.9 to 6.3±1.2 Hz (*P*<0.05). In four of five neurones mean amplitudes increased slightly although there was no significant change in mean (11.0±1.6 versus 11.8±1.9 pA). The decay time of sEPSCs, unlike that of sIPSCs was also unaltered (6.7±0.7 versus 6.5±0.6 ms). To obtain an overall estimate of the change in excitation resulting from spontaneous glutamate release we calculated total excitatory charge transfer. In control conditions this was 34.1±7.5 pC and in the presence of tiagabine it was 68.8±12.6 pC (*P*<0.05), an increase of 115±36%.

Thus, despite the relatively complex effects of tiagabine in patch clamp studies, the overall changes in charge transfer paralleled the results with VmD estimates, with both excitation and inhibition. The increase in I:E ratio noted in VmD studies was accompanied by similar changes in excitability noted for the other AED tested (Fig. 4B). Thus, spike threshold was raised from 18.8±0.5 to 24.9±0.5 mV (*P*<0.05), and the number of spikes generated during depolarization fell substantially from 3.5±0.4 to 1.3±0.21 (*P*<0.05). As with the other drugs, action potential amplitude (97.9±2.8 versus 99.4±2.3 mV) and half-width (0.33±0.03 ms versus 0.38±0.03 mV) were unaffected.

### Other drugs

It is clear from the above studies that clinically effective AEDs consistently increase the ratio of global background synaptic inhibition to excitation and simultaneously decrease neuronal excitability. The consistency of these effects raises the question as to whether these effects may simply be artefacts of the novel experimental approach, and have no relevance to clinical therapeutic actions. As a way of controlling for this we have tested a number of drugs that do not exhibit clinical AED activity.

#### PDC

**Fig. 5:**
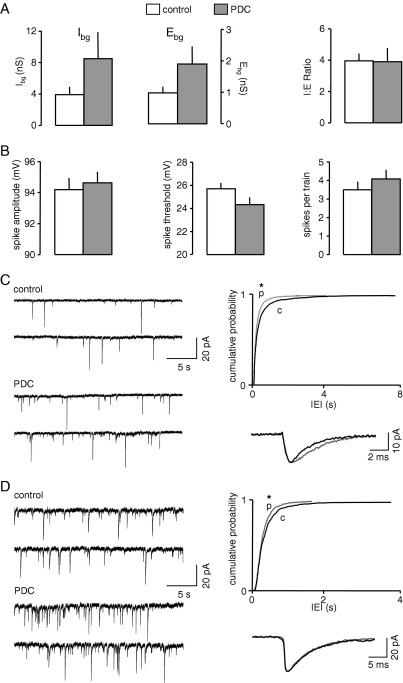
Summary of VmD and whole cell patch experiments with PDC. (A) Like tiagabine, the glutamate uptake blocker increased both background conductances. However the much lesser effect on I_bg_ meant that there was no overall change in I:E ratio. (B) There was little concurrent change in excitability. (C) Recordings of sEPSCs show that the drug primarily increased the frequency of events and prolonged their decay time. (D) PDC also increased the frequency of sIPSCs but did not alter decay time. The asterisks indicate significant differences compared to control values. Asterisks indicate a significant difference compared to control values (*P*<0.01, KS).

As a like-for-like comparison with tiagabine we have examined the effect of a glutamate uptake blocker, PDC (100 μM) in both VmD and whole cell patch configurations. In six neurones, E_bg_ was increased by PDC from 1.0±0.2 to 2.0±0.5 nS (*P*<0.05). Concurrently, I_bg_ also increased (3.9±1.0 to 8.5±3.5 nS). The parallel rise in both conductances resulted in little overall change in I:E ratio, which was 4.0±0.5 in control, and 3.9±0.9 in the presence of PDC (96±19%). The data are summarized in Fig. 5A.

Whole cell patch clamp recordings revealed a number of effects of PDC on both sEPSCs and sIPSCs. There was a clear effect on the frequency of sEPSCs (Fig. 5C, *n*=6). IEI decreased from 591±243 to 345±129 ms (*P*<0.01, KS Fig. 5C) during perfusion with PDC reflecting an increase in frequency from 3.3±1 to 4.7±1.1 Hz (*P*<0.05, paired *t*-test). Although amplitude was unaltered (19.5±4.0 versus 19.8±3.2 pA), total decay time increased from 7.1±0.6 ms to 8.4±0.9 ms (*P*<0.05, paired *t*-test). This is illustrated by the averaged sEPSCs from one neurone shown in Fig. 5C. Rise time was unaltered (2.5±0.5 versus 2.3±0.3 ms), but the change in frequency and decay time combined to induce a significant increase in total charge transfer from 60.2±22.4 to 101.5±30.0 pC (*P*<0.05, paired *t*-test), a rise of 93±24%.

Blocking glutamate uptake also altered spontaneous GABA release (*n*=6) and the data are illustrated in Fig. 5D. A significant (*P*<0.01, KS) decrease in IEI (from 110±16 to 90±11 ms) reflected an average increase in sIPSC frequency to 122±9% of control in the presence of PDC (10.1±1.5 to 12.1±1.5 Hz, *P*<0.05, paired *t*-test). Additionally, mean amplitude increased from 22.3±1.4 to 27.5±2.8 pA, although this was not significant. Total decay time was slightly, but not significantly, longer (12.9±1.0 versus 13.8±1.5 ms). Overall, the change in sIPSC parameters resulted in a rise in total charge transfer to 166±25% of control levels in PDC (435.5±113.6 versus 637.7±126.7 pC, *P*<0.05, paired *t*-test).

Thus, the effects of PDC on sEPSCs and sIPSCs are in concordance with data from VmD studies, which show a relatively balanced increase in global background inhibition and excitation. These changes were not accompanied by any clear alteration of neuronal excitability (Fig. 5B). Thus, spike thresholds were 25.7±20.5 and 24.3±0.6 mV in the absence and presence of PDC, respectively. Spike amplitude was unchanged (94.2±0.7 versus 94.6±0.7 mV) as was the number of spikes elicited by a depolarizing pulse (3.5±0.4 versus 4.1±0.5).

#### Clozapine

Clozapine is an atypical antipsychotic drug effective against both negative and positive symptoms of schizophrenia. One side effect that has long been known is promotion of seizures (see Wong and Delva, 2007). We have used the VmD approach to determine if it can alter I:E ratio perhaps leading to increased neuronal excitability and proconvulsant effects. In four neurones, E_bg_ was 2.2±0.6 nS, and this was essentially unaltered (2.5±0.7 nS) in the presence of clozapine (1 μM). I_bg_ was also unaffected (5.1±1.1 versus 5.9±1.4 nS) so that I:E ratio showed little change (3.2±0.5 versus 3.6±0.4). Concurrently there was little change in spike threshold (18.7±2.1 versus 18.9±2.0 mV) or number of spikes evoked by a long depolarizing pulse (3.9±0.1 versus 3.9±0.2).

#### Fluoxetine

There has been some controversy over whether antidepressants, including the selective 5HT uptake blocker, fluoxetine, may have pro- or anticonvulsant effects (see Jobe and Browning, 2005). We have examined the acute effects of fluoxetine (10 μM) in VmD experiments (*n*=4). As with clozapine, fluoxetine induced no significant change in either E_bg_ (although slightly increased to 115±6% of control) or I_bg_ (94±6%), and consequently, I:E ratio (97±7%). The lack of change in background conductances was congruent with an unaltered excitability (spike threshold 21.4±2.7 versus 19.6±2.9 mV: number of spikes 4.5±0.6 versus 3.8±0.7). Similar data were obtained with another antidepressant, desipramine (not shown).

### Effects of convulsant treatments

Given the consistent changes in ratio of background inhibition to excitation noted with a range of AED, it was of interest to determine what effect treatments that provoke synchronized epileptiform activity would have. To this end we examined the effects of the GABA_A_-antagonist, bicuculline, reduction of [Mg^2+^]_o_, and the transient A-type K^+^-channel blocker, 4-aminopyridine (4-AP). All three treatments resulted in the induction of spontaneous, recurrent epileptiform activity. Once this stage was reached, it was extremely difficult to obtain the stable membrane potential required for VmD estimates, so the data below were mostly gathered in the period before epileptiform activity was initiated. When the initial rate of discharges was low, we were able to continue measurements for a short period following this.

#### Bicuculline

**Fig. 6:**
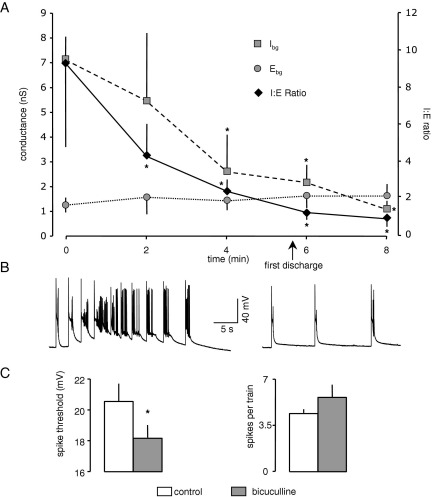
Background synaptic conductances during blockade of GABA_A_r. The graph in (A) shows the time course of the effects of bicuculline (10 μM; *n*=5), which reached the recording chamber at time zero. The drug induced a substantial and progressive decrease in I_bg_ with a concurrent weak increase in E_bg_. This resulted in a fall in I:E ratio to the point were the conductances were approximately equal. At this stage synchronous epileptiform discharges became apparent. These usually presented as an initial a prolonged event, which was succeeded by regular recurrent discharges that were much briefer (B). (C) Shows that cellular excitability was increased at time close to when epileptiform discharges became apparent. Asterisks indicate a significant difference compared to control values (*P*<0.05, paired *t*-test).

We previously reported the effects of bicuculline on global inhibition and excitation at a single time point (Greenhill and Jones, 2007). In the current studies we have followed the time course of changes in I_bg_ and E_bg_ during perfusion with the blocker. Summary data for five neurones are shown in Fig. 6A. It is clear that there was a rapid and progressive decrease in I_bg_ in the presence of the GABA-antagonist. At the same time, E_bg_ gradually rose although at no point did this reach significance. These combined changes resulted in a precipitous decline in I:E ratio. After 8 min the ratio was around 1:1 from a starting point of over 9:1. Also shown on the time course graphs in Fig. 6, is the mean point at which spontaneous epileptiform events became apparent. In most cases the initial event was a long-lasting ictal-like discharge and was immediately followed by regular, brief (150–450 ms duration) interictal-like events (Fig. 6B), which have been described previously (e.g. Jones, 1988; Jones and Lambert, 1990a). At this stage, I:E ratio was approaching unity and cellular excitability was increased (Fig. 6C), with spike threshold lower (20.6±1.1 versus 18.2±0.9 mV) and the number of spikes evoked by a depolarizing pulse elevated (4.3±0.3 to 5.5±1.0 mV).

#### Low-Mg^2+^

**Fig. 7:**
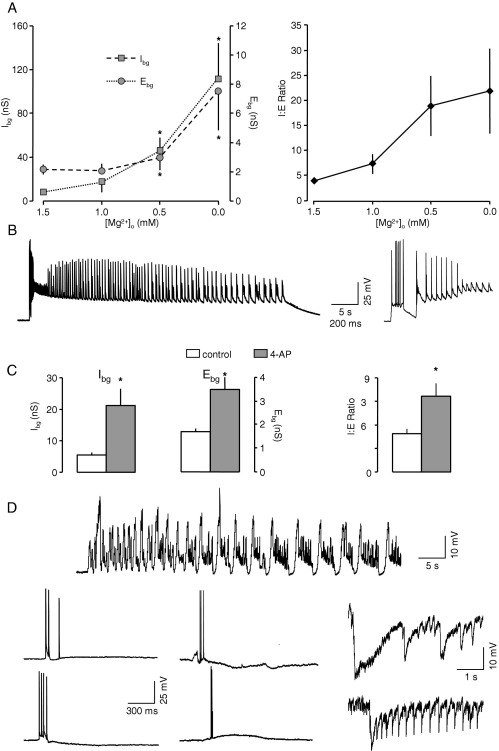
Changes in background synaptic conductances elicited by other convulsants. (A) Successively reducing [Mg^2+^]_o_ from 1.5 to 0 mM (*n*=6) resulted in a substantial rise in E_bg_, but a dramatic increase in I_bg_, resulting in a progressive increase in I:E ratio. At 0 mM, long lasting recurrent epileptiform events began to occur. One such event is shown in (B), with an expanded trace of the initial part of the event also shown. (C) Perfusion with the K-channel blocker, 4-AP (*n*=6) also resulted in a rise in both E_bg_ and I_bg_, although the latter effect was considerably less marked than that seen with lowering [Mg^2+^]_o_. Nevertheless, a rise in I:E ratio was also recorded. (D) Synchronized discharges appeared in all neurones after 12–15 min. These were a mixture of ictal-like events, briefer discharges, which were complex in nature consisting of ectopic spikes, depolarizing and hyperpolarizing components, and also large synchronized inhibitory events, with repetitive, inhibitory afterdischarges. Asterisks indicate a significant difference compared to control values (*P*<0.05, paired *t*-test).

The epileptogenic effects of lowering [Mg^2+^]_o_ in the EC have been described in detail previously (Walther et al., 1986; Jones and Heinemann, 1988; Jones and Lambert, 1990b). We examined the effects of progressively reducing [Mg^2+^]_o_ from 1.5 to 1.0, 0.5 and then nominally 0 mM in six neurones. The results are summarized in Fig. 7A. In control conditions (1.5 mM) mean E_bg_ was 2.2±0.4 nS and I_bg_ was 8.0±1.3 nS. The most obvious effect of a reduction of Mg^2+^to 1.0 mM was a doubling of I_bg_ with no effect on E_bg_. At 0.5 mM I_bg_ was increased by over 500%, and now E_bg_ was also increased, but only by around 50% of its control value. The biggest change in E_bg_ occurred on switching to 0 mM Mg^2+^, when an increase of over 350% rapidly occurred. However, at this stage, I_bg_ was also further increased by over 1000%. All told, the relative changes in excitation and inhibition resulted in a ratio change from 3.8±0.3 to 21.8±8.5 in favour of inhibition. At 1.0 mM, two out of six neurones began to display spontaneous slow wave oscillations as described by us previously (Cunningham et al., 2006). However, within 5–8 min of reducing Mg^2+^ to zero, all neurones began displaying a brief interictal like events, which were rapidly followed by development of long lasting (10's of s to minutes) ictal-like events (Fig. 7B), precluding any further VmD estimates. Immediately prior to the development of epileptiform activity excitability increased with spike threshold falling from 22.3±1.3 to 16.2±0.9 mV, although surprisingly the number of spikes evoked by a suprathreshold pulse was unaltered.

#### 4-AP

4-AP has frequently been used as an acute convulsant in the entorhinal cortex (e.g. Barbarosie et al., 2000). We have tested its effects on background synaptic conductances in six neurones. We tested both 20 and 40 μM, but there appeared to be little difference so results were pooled. The results are summarized in Fig. 7C. As with lowered-Mg^2+^, 4-AP resulted a substantial increase in both E_bg_ (from 1.7±0.2 to 3.5±0.5 nS; *P*<0.05) and I_bg_ (6.1±0.8 versus 23.5±5.8 nS; *P*<0.05). Overall, the changes were not as marked as with low-Mg^2+^ and the I:E ratio was increased from 3.7±0.4 to 7.2±1.5. Again, VmD estimates were made just prior to or after the first appearance of epileptiform discharges. The latter were complex and variable and consisted of combination of brief interical like events and longer lasting ictal-like discharges as well as spontaneous ectopic spikes, synchronized inhibitory discharges and combinations of these (Fig. 7D). At this time, intrinsic excitability appeared largely unaffected, with spike threshold at 23.4±1.0 mV compared to the control value of 21.8±1.9 mV and number of spikes on a suprathreshold pulse 4.8±0.2 compared to 5.5±0.2. As with bicuculline and low-Mg^2+^, the occurrence of recurrent spontaneous epileptiform activity made VmD estimates almost impossible.

## Discussion

The traditional view of epileptogenesis is that it derives from a disrupted balance between neuronal inhibition and excitation, manifest at the intrinsic (membrane ion channels) and/or synaptic level. A huge body of research has layered complex and interacting facets of ion channel behaviour and synaptic communication into this simplistic concept. Derivation of the complexity of intrinsic/synaptic network changes associated with epileptogenesis has been substantially informed by experimental investigation of dysfunction of behaviour in the pathological condition, and by the determination of the pharmacological actions of drugs used to treat it. Nevertheless, the basic tenet that epileptogenesis is a consequence of excess excitation, deficient inhibition, or a combination of the two, is still widely accepted.

AED have been generally categorized as those that primarily aim to promote inhibition, reduce excitation, or modify voltage gated ion channels. It is also the case that identified gene mutations, which are emerging from molecular genetic studies of epileptic syndromes, are beginning to fall into similar broad categories (see Rogawski and Loscher, 2004). However, it is accepted that AED are promiscuous in their actions, and most have multiple targets. In the current study, rather than focus on specific molecular targets, we have attempted to determine whether diverse drugs may elicit a generalized change in network excitability that could underlie anticonvulsive actions. The approach we used was based on a method of estimating global synaptic conductances from membrane potential fluctuations arising as a result of spontaneous firing and transmitter release in presynaptic inputs (Rudolph et al., 2004). It allows a simultaneous estimation of the strength of background inhibition mediated by GABA_A_r and excitation mediated by AMPAr, independent of the kinetics or site of origin of the individual synaptic activities. However, while it allows us to integrate overall synaptic responses it does not allow us to distinguish between fast and slow inhibition mediated at somatic and dendritic sites for example. These may have very different roles and function in epileptogenesis (e.g. Maglóczky and Freund, 2005; Knopp et al., 2008). It also does not take account of background conductances mediated by GABA_B_ or NMDAr, for example, but this is unlikely to be a problem since neither appears to contribute much to spontaneous activity in the EC (e.g. Berretta and Jones, 1996; Bailey et al., 2004). Non-synaptic neuronal communication via gap-junctions is also important in epilepsy (e.g. Traub et al., 2004; Nemani and Binder, 2005) and these are also not accounted for directly by VmD. Nevertheless, we have previously validated the use of this approach to estimate these conductances in EC neurones (Greenhill and Jones, 2007a). Despite the limitations, the approach provides an estimation of background inhibition and excitation (Rudolph et al., 2004), and our previous studies have shown that the background conductances are altered in a predictable and explicable way by drugs with known pharmacological actions (Greenhill and Jones, 2007a). Thus, we feel confident in applying the VmD approach to determining the global effects of AED in the balance between inhibition and excitation in cortical networks.

The most striking observation from these studies is the consistent effect of all the AEDs studied in increasing the I:E ratio in favour of I_bg_. This applied regardless of the effect of each drug on individual conductances. For example, phenytoin and lamotrigine both increased I_bg_ and decreased E_bg_, whereas gabapentin and felbamate decrease E_bg_ with little effect on I_bg_, and tiagabine increased both. Nevertheless, in each case, I:E ratio was increased likely reflecting an overall strengthening of inhibition over excitation. There did appear to be quantitative differences in the extent of the increased ratio. Perhaps unsurprisingly, the biggest changes were seen with phenytoin and lamotrigine, which induced changes in I_bg_ and E_bg_ in opposite directions. However, the consistency of the effect among the AED tested could well point to this as common mechanism underlying anticonvulsant effects.

The changes in background synaptic activity were accompanied by a reduction in cellular excitability, evidenced by decreased spike threshold and depolarization induced firing. Again, these changes were consistent among the AED tested and it is tempting to link the excitability change with the relative increase in background inhibition. Increasing evidence has suggested that spontaneous transmitter release and the resultant synaptic noise are an important determinant of neuronal excitability (Pare et al., 1998; Stevens and Zador, 1998; Destexhe and Pare, 1999; Ho and Destexhe, 2000; Rudolph and Destexhe, 2001; Stacey and Durand, 2000; Fellous et al., 2003; Shu et al., 2003a) so it is conceivable that the decline in excitability may be associated with the overall increase in synaptic inhibition. However, we have no direct evidence that this is the case at present, and the link remains circumstantial.

We also studied several drugs that might not be expected to exert anticonvulsant effects. Glutamate uptake appears to be compromised in a number of models of epilepsy (e.g. Gorter et al., 2002; Campbell and Hablitz, 2008), mice lacking glutamate transporters display an epileptic phenotype (e.g. Tanaka et al., 1997), and in some epilepsies, mutations encoding genes for glutamate transporters may predispose towards epilepsy (e.g. de Vries et al., 2009). Thus, blocking glutamate uptake might be predicted to have proconvulsant effects. In whole cell patch clamp recordings the glutamate uptake blocker, PDC, increased the frequency of sEPSCs and slightly prolonged their decay times, but was without effect on amplitude. It also increased the frequency of sIPSCs, and caused a non-significant increase in amplitude without effect on decay times. These effects are partly due to increased access of released glutamate to presynaptic kainate receptors on excitatory terminals and both terminals and soma/dendrites of inhibitory neurones (Chamberlain and Jones, unpublished observations). Overall, these changes resulted in an increase in total charge transfer associated with both spontaneous glutamate and GABA release, and these changes were mirrored in VmD studies. Thus, although PDC increased E_bg_ it also promoted I_bg_, with the result that there was no change in I:E ratio. Significantly, PDC also failed to change neuronal excitability, further supporting the possibility that excitability and I:E ratio may be linked.

There is considerable controversy over the role of monoaminergic systems in epilepsy and, while there is a large body of evidence to suggest antidepressants are proconvulsant, there are also clear indications for anticonvulsant effects (see Jobe and Browning, 2005). Our studies do not provide evidence to really support either viewpoint, since we found that the antidepressant drugs, fluoxetine and desipramine, failed to alter either I_bg_, E_bg_, or intrinsic neuronal excitability. Finally, there is evidence to suggest that the atypical antipsychotic, clozapine, may have proconvulsant effects (Wong and Delva, 2007). Despite evidence from studies in prefrontal cortex to suggest that clozapine can potentiate (Chen and Yang, 2002) or inhibit excitation (Gao, 2007) and enhance inhibition (Gao, 2007), in our studies, it failed to change any parameters estimated in VmD experiments.

So overall, of those agents we have tested to date, only drugs with unequivocal clinical anticonvulsant activity elicited the increased I:E ratio and decreased neuronal excitability. These effects occurred regardless of the known molecular targets of the diverse agents. It is also clear that the effects on global conductances correlated reasonably well with data on spontaneous glutamate and GABA release from whole-cell patch clamp recordings. We have shown in previous experiments that AED alter release in different ways and by different mechanisms (Cunningham and Jones, 2000; Cunningham et al., 2000, 2003, 2004; Yang et al., 2007). We have extended these studies here, and some discussion is warranted.

Previously, we have shown that felbamate can decrease sEPSC frequency by blocking presynaptic facilitatory NMDA autoreceptors (Yang et al., 2007). In contrast, although felbamate has been suggested to enhance GABA currents in cultured neurones (Kume et al., 1996; Rho et al., 1997; Simeone et al., 2006) we have now shown that the drug has little effect on sIPSCs at EC synapses, although it slightly, but non-significantly increased decay time. The lack of effect could be associated with a demonstrated sub-unit specificity of felbamate at recombinant GABA_A_r (Simeone et al., 2006). Thus, the patch clamp studies are in agreement with the lack of effect on I_bg_.

Qualitatively, gabapentin resembled felbamate in its action. We reported earlier that gabapentin acts presynaptically to reduce the frequency of sEPSCs in EC neurones, an effect due, at least partly, to reduced Ca-influx via P/Q-type VGCC (Cunningham et al., 2004). We show here, that gabapentin has little effect on frequency, amplitude or kinetics of sIPSCs, again in agreement with our VmD studies. There is reasonable evidence that prolonged application of gabapentin may enhance GABA levels and turnover in some brain regions (see White et al., 2007), but this is not reflected in our acute studies. In support of this, Takasu et al. (2008) found no effect of gabapentin on sIPSCs in rat locus coeruleus neurones under normal conditions. Experiments in synaptosomes prepared from human neocortex showed a decrease in depolarization induced GABA release by gabapentin, but there was no effect on basal release (Brawek et al., 2009). Finally, Parker et al. (2004) showed that gabapentin reduced release of glutamate from rat neocortical slices, while having no effect on GABA. They suggest that this is due to differential activation of presynaptic GABA_B_ auto and heteroreceptors, but whatever the mechanism their results are in excellent concordance with ours from both patch clamp and VmD analysis.

Tiagabine had effects on both sIPSCs and sEPSCs in whole cell patch clamp studies. The prolongation of sIPSCs presumably reflects the blockade of GABA uptake, and is similar to effects on sIPSCs reported in other areas (Williams et al., 1998; Chan and Yung, 1999, 2003). This was accompanied by a decrease in frequency, which again has been reported previously (Williams et al., 1998; Chan and Yung, 1999, 2003), and is likely to be mediated by activation of presynaptic GABA_B_ autoreceptors as a consequence of increased accumulation of GABA following uptake blockade (Chan and Yung, 1999, 2003). In contrast to these previous studies where amplitude was either unaltered (Williams et al., 1998), or reduced (Chan and Yung, 1999, 2003), sIPSC amplitude was increased by tiagabine at EC synapses. This could suggest that postsynaptic GABA_A_r are not saturated during spontaneous release of GABA at these synapses. In contrast to sIPSCs, the amplitude and decay time of sEPSCs was not significantly affected by tiagabine, so it seems unlikely that the drug (at the concentration tested) has an effect on glutamate uptake (cf PDC) at these synapses. However, tiagabine did induce a significant increase in frequency of sEPSCs. We have no ready explanation for this at present. Activation of presynaptic GABA_A_r have been shown to enhance glutamate release at terminals on neurones at a number of sites (Koga et al., 2005; Jang et al., 2006; Alle and Geiger, 2007; Stell et al., 2007) so one possibility is that of spillover of GABA to GABA_A_r on glutamate terminals occurs following uptake blockade with tiagabine. Whatever the mechanisms involved, tiagabine clearly had complex effects on different parameters of both spontaneous glutamate and GABA release. However, when we calculated total charge transfer associated with sEPSCs and sIPSCs, overall, both were increased by tiagabine, with a more dominant effect on inhibition. Again, this is in good agreement with the VmD estimates, which suggest a doubling of I:E ratio and accompanying decline in cellular excitability.

Finally, given the consistency with which the AED altered global background synaptic activity, we thought it was worthwhile testing the effects of agents known to provoke acute synchronized epileptiform activity. The simplistic prediction would be that these might be expected to provoke opposite effects to AED. This appeared to be case with the GABA_A_r antagonist, bicuculline. Thus, I_bg_ was dramatically reduced, as expected. At the same time, E_bg_ was slightly increased, which we attribute to loss of network inhibition of the principal cells and increased recurrent excitation (Dhillon and Jones, 2000). When I:E ratio approached unity, neuronal excitability was increased and spontaneous epileptiform activity was initiated.

The situation with the other convulsant treatments was much more complicated. Both strongly increased E_bg_ as might be predicted, but both also strongly increased I_bg_ and I:E ratio. The increase in inhibition is likely to be due to a strong enhancement of GABA release. We have previously shown that both inhibition and excitation are increased during the development of epileptiform activity in Mg-free medium (Jones, 1994). This could occur by a number of mechanisms. Lowering [Mg^2+^]_o_ is likely to remove a physiological block of presynaptic VGCC leading to increased release (Carbone et al., 1997). In addition, it will increase excitability of interneurones via reduced surface charge screening (see Mangan and Kapur, 2004). Interneurones in the EC receive a very powerful network drive mediated by NMDAr (Jones and Buhl, 1993) and this should be increased by removal of the Mg voltage-dependent block of these receptors. GABA-release is also facilitated by presynaptic NMDAr (Woodhall et al., 2001), and this effect would be increased by loss of the Mg-dependent block. Of course, all these considerations will also apply to the glutamatergic principal cells.

4-AP could also be predicted to increase both excitation and inhibition. Indeed, synchronized GABAergic potentials are a feature of 4-AP-induced epileptiform activity. Again this would arise partly through increased release of both transmitters as a result of blockade of repolarizing K-currents in presynaptic terminals (Southan and Robertson, 2000; Qian and Noebels, 2005; Westphalen and Hemmings, 2006). It is also likely to lead to increased neuronal excitability in some neuronal subtypes, with consequent further effects for network driven release. It is clearly the case that the network effects of 4-AP are extremely complex with combinations of synchronized excitation, inhibition and generation of ectopic spikes all evident during epileptiform activity (see also de Guzman et al., 2004; Keros and Hablitz, 2005).

The mechanisms underlying synchronicity in the low-Mg and 4-AP models, therefore, do not fit simply with the decreased I:E ratio model that is apparent with bicuculline. In truth, this is not particularly surprising. The complexity and multiplicity of effects of these treatments largely precludes the simple approach afforded by the VmD method. VmD measurements are limited to estimating the global effects mediated by GABA_A_r and AMPAr. They do not take any account of background excitation mediated by either NMDAr or inhibition mediated by GABA_B_r (Rudolph et al., 2004) both of which are likely to occur during increased release of transmitters induced by low-Mg or 4-AP (e.g. Jones, 1994). VmD does not take account, either, of altered intrinsic membrane conductances (Rudolph et al., 2004).

It should also be noted that the activity elicited by any of these treatments is not true epileptic activity in any case, but an artificial provocation of synchronization; the term epileptiform is used advisedly. The disruption of normal function is induced by multiple changes in both synaptic and intrinsic properties of neurones interacting across the whole network. The very different forms of discharge evoked by the three convulsant treatments are testament to different underlying processes. Whilst the effects of these convulsants may not realistically reflect the changes associated with true pathological epilepsies, at the same time it is unlikely that a simple imbalance in inhibition and excitation is the pathological cause of epilepsy, and indeed different epilepsies will certainly have different underlying pathological causes. It is tempting to suggest that our results indicate that AED reduce seizures in a “symptomatic” way without targeting specific core deficits in network function. In this scenario, drug-resistance could involve core deficits that would not be ameliorated by a simple shift in I:E balance, or were not dependent on such a change. To date we have only assessed the effects of the AEDs against “normal” network function, so now there is a need to expand these studies to determine if VmD can be of value in assessing changes in network function in chronic epileptic tissue (e.g. from kindled rats or pilocarpine/kainate models), and whether the effects of the AED may differ in this situation.

## Conclusion

Thus, in summary, we have found that a common effect of AED is to elicit a shift in the ratio of global inhibition to global excitation in favour of the former, regardless of the change in the individual conductances. This shift occurs concurrently with a decline in intrinsic neuronal excitability, although any link between the two is circumstantial at present. Nevertheless, it may be that the specific molecular targets of individual drugs are less important than the ability to globally shift neuronal and network excitability to a more stable configuration. To date, we have only recorded these effects in normal tissue, and the situation may differ in the chronic epileptic condition. Of course, in the chronic condition, there are will be alterations to network activity *per se*. What we need to do now is examine the effects of the AED on global conductances in the hyperexcitable state that characterizes the pathological condition, to determine if the alterations in I:E ratio are still evident, and correlated with amelioration of synchronized discharges.
